# Bioelectrical impedance phase angle as a prognostic indicator in breast cancer

**DOI:** 10.1186/1471-2407-8-249

**Published:** 2008-08-27

**Authors:** Digant Gupta, Carolyn A Lammersfeld, Pankaj G Vashi, Jessica King, Sadie L Dahlk, James F Grutsch, Christopher G Lis

**Affiliations:** 1 Cancer Treatment Centers of America ® (CTCA) at Midwestern Regional Medical Center, Zion, IL, USA

## Abstract

**Background:**

Bioelectrical impedance analysis (BIA) is an easy-to-use, non-invasive, and reproducible technique to evaluate changes in body composition and nutritional status. Phase angle, determined by bioelectrical impedance analysis (BIA), detects changes in tissue electrical properties and has been hypothesized to be a marker of malnutrition. Since malnutrition can be found in patients with breast cancer, we investigated the prognostic role of phase angle in breast cancer.

**Methods:**

We evaluated a case series of 259 histologically confirmed breast cancer patients treated at Cancer Treatment Centers of America. Kaplan Meier method was used to calculate survival. Cox proportional hazard models were constructed to evaluate the prognostic effect of phase angle independent of stage at diagnosis and prior treatment history. Survival was calculated as the time interval between the date of first patient visit to the hospital and the date of death from any cause or date of last contact/last known to be alive.

**Results:**

Of 259 patients, 81 were newly diagnosed at our hospital while 178 had received prior treatment elsewhere. 56 had stage I disease at diagnosis, 110 had stage II, 46 had stage III and 34 had stage IV. The median age at diagnosis was 49 years (range 25 – 74 years). The median phase angle score was 5.6 (range = 1.5 – 8.9). Patients with phase angle <= 5.6 had a median survival of 23.1 months (95% CI: 14.2 to 31.9; n = 129), while those > 5.6 had 49.9 months (95% CI: 35.6 to 77.8; n = 130); the difference being statistically significant (p = 0.031). Multivariate Cox modeling, after adjusting for stage at diagnosis and prior treatment history found that every one unit increase in phase angle score was associated with a relative risk of 0.82 (95% CI: 0.68 to 0.99, P = 0.041). Stage at diagnosis (p = 0.006) and prior treatment history (p = 0.001) were also predictive of survival independent of each other and phase angle.

**Conclusion:**

This study demonstrates that BIA-derived phase angle is an independent prognostic indicator in patients with breast cancer. Nutritional interventions targeted at improving phase angle could potentially lead to an improved survival in patients with breast cancer.

## Background

In the United States, breast cancer is the most common non-skin cancer and the second leading cause of cancer-related death in women [1].

Malnutrition is a frequent manifestation in patients with advanced cancer and is a major contributor to morbidity and mortality [2]. Malnutrition is characterized by changes in cellular membrane integrity and alterations in fluid balance [3]. As a result, measurement of body composition is an important component of overall nutritional evaluation in cancer patients [4-6].

Several studies have investigated the relationship between diet, physical activity, obesity and survival in breast cancer [7-11]. A prospective study was performed on 1,490 women diagnosed and treated for early-stage breast cancer between 1991 and 2000. In univariate analysis, reduced mortality was weakly associated with higher vegetable-fruit consumption, increased physical activity, and a body mass index that was neither low weight nor obese [7]. Another study investigated the influence of diet, including dietary fat (percentage energy), fiber, vegetable, and fruit intakes, and micronutrients (folate, carotenoids, and vitamin C) on overall survival in 516 postmenopausal women diagnosed with breast cancer. In the multivariate analysis, the hazard ratio of dying in the highest tertile compared to the lowest tertile of total fat, fiber, vegetable, and fruit was 3.12 (95% CI = 1.79–5.44), 0.48 (95% CI = 0.27–0.86), 0.57 (95% CI = 0.35–0.94), and 0.63 (95% CI = 0.38–1.05), respectively (P <or= 0.05 for trend, except for fruit intake) [9]. A review article summarized the evidence from clinical and epidemiologic studies that have examined the relationship between nutritional factors, survival, and recurrence after the diagnosis of breast cancer. The article reported that overweight or obesity was associated with poorer prognosis in the majority of the studies that have examined this relationship. Treatment-related weight gain also may influence disease-free survival, reduce quality of life, and increase risk for comorbid conditions [11]. Clearly, much remains to be learned about the role of nutritional factors in survival after the diagnosis of breast cancer, especially with the advent of novel techniques to assess nutritional status.

Historically, nutritional status has been evaluated by various objective measures, including anthropometric (e.g. weight change, arm muscle circumference, triceps skinfold thickness) and laboratory (serum albumin, transferrin assays and nitrogen balance studies) measurements. Anthropometric methods are not ideal in a clinical setting because they are time-consuming and require well-trained staff. Some of the objective measures such as serum albumin are likely to be influenced by many non-nutritional factors [12-15]. Furthermore, some objective indicators such as serum albumin have long half-lives, thus, assessing changes in the nutritional status over a short period of time is challenging. A less common tool to assess body composition and nutritional status, called Bioelectrical Impedance Analysis (BIA), can overcome some of these challenges. BIA is an easy-to-use, non-invasive, and reproducible technique to evaluate changes in body composition.

BIA has been validated for the assessment of body composition and nutritional status in a variety of patient populations including cancer [2,5,16-26]. BIA measures body component resistance (R) and reactance (Xc) by recording a voltage drop in applied current [27]. Resistance is the restriction to the flow of an electric current, primarily related to the amount of water present in the tissues. Reactance is the resistive effect produced by the tissue interfaces and cell membranes [28]. Reactance causes the current to lag behind the voltage creating a phase shift, which is quantified geometrically as the angular transformation of the ratio of reactance to resistance, or the phase angle [29].

Phase angle reflects the relative contributions of fluid (resistance) and cellular membranes (reactance) of the human body. By definition, phase angle is positively associated with reactance and negatively associated with resistance [29]. Lower phase angles suggest cell death or decreased cell integrity, while higher phase angles suggest large quantities of intact cell membranes [30]. Phase angle has been found to be a prognostic marker in several clinical conditions such as human immunodeficiency virus infection, liver cirrhosis, chronic obstructive pulmonary disease, hemodialysis, sepsis, lung cancer [30-35]. Previously, we had demonstrated the prognostic role of phase angle in advanced colorectal and pancreatic cancer [36,37]. The primary objective of this study, which builds upon our prior research work in this area, was to evaluate the association of BIA-derived phase angle with survival in patients with breast cancer.

## Methods

A retrospective chart review was performed on a consecutive case series of 259 female breast cancer patients treated at Cancer Treatment Centers of America (CTCA)^® ^at Midwestern Regional Medical Center (MRMC) between January 2001 and May 2006. The patients were identified from the MRMC tumor registry. Only patients with a histologically confirmed diagnosis of breast cancer were included in this study. The study was approved by the Institutional Review Board at MRMC.

All patients underwent a baseline nutritional assessment, which included laboratory measurements of serum albumin, prealbumin and transferrin, subjective global assessment (SGA), and BIA. BIA was performed by a registered dietitian using a Bioelectrical Impedance Analyzer, Model BIA-101Q: RJL Systems, Clinton Township, MI, USA. BIA was conducted while patients were lying supine on a bed or exam table, with legs apart and arms not touching the torso. All evaluations were conducted on the patients' right side using the four surface standard electrode (tetra polar) technique on the hand and foot [23]. Resistance (R) and reactance (Xc) were directly measured in Ohms at 50 Khz, 800 μA using RJL BIA. One assessment of resistance (R) and reactance (Xc) was made. Phase angle was calculated using the following equation: Phase Angle = (Resistance/Reactance)*(180/π).

All data were analyzed using SPSS 11.5 (SPSS Inc., Chicago, IL, USA). Patient survival was defined as the time interval between date of first patient visit to the hospital and date of death from any cause or date of last contact/last known to be alive. The Kaplan-Meier or product-limit method was used to calculate survival. The log rank test statistic was used to evaluate the equality of survival distributions across different strata. A difference was considered to be statistically significant if the p value was less than or equal to 0.05. Survival was also evaluated using univariate and multivariate Cox regression analysis. Variables evaluated included phase angle, age at diagnosis, prior treatment history and stage at diagnosis. For the purpose of univariate analysis, phase angle measurements were categorized using SPSS into 2 mutually exclusive groups with median = 5.6 as the cut-off. For the purpose of multivariate analyses, phase angle was treated as a continuous variable. Similarly, stage at diagnosis variable was treated as a dichotomous variable with 2 categories – early stage (stages I and II) and late stage (stages III and IV).

## Results

At the time of this analysis (May 07), 85 patients had expired and 174 were censored, as shown in Table 1. The cut-off date for the follow-up for all participants was May 07. The median age at diagnosis was 49 years (range 25 – 74 years). The median phase angle score was 5.6 (range = 1.5 – 8.9). Phase angle was found to be non-normally distributed. Table 2 shows the univariate survival analysis of different prognostic factors. Phase angle, tumor stage and treatment history were found to be statistically significantly associated with survival.

**Table 1:** Patient characteristics

**Characteristic**	**Categories**	**Number**	**Percent (%)**
Vital Status	Expired	85	32.8
	Censored^1^	174	67.2
Prior Treatment	Progressive disease	178	68.7
History	Newly diagnosed	81	31.3
Stage at Diagnosis	Stage I	56	21.6
	Stage II	110	42.5
	Stage III	46	17.8
	Stage IV	34	13.1
	Missing	13	5.0
Age at Diagnosis	25–35	26	10
	36–45	72	27.8
	46–55	88	33.9
	56–65	66	25.6
	66–75	7	2.7

^1 ^Patients who reached the end of their follow-up without experiencing death.

N = 259

**Table 2:** Univariate Kaplan-Meier survival analysis

**Variable**	**Median survival in months**	**Log-rank score**	**P-value**
Phase Angle			
• <= 5.6	23.1 (14.2 to 31.9)	4.9	0.031
• >5.6	49.9 (35.6 to 77.8)		
Tumor Stage			
• Stage I and II	54.2 (35.3 to 83.2)	5.4	0.021
• Stage III and IV	22.9 (14.0 to 31.9)		
Treatment History			
• Newly diagnosed	54.6 (53.8 to 55.4)	50.2	0.0001
• Progressive disease	18.7 (14.8 to 22.5)		

N = 259

**Figure 1:**
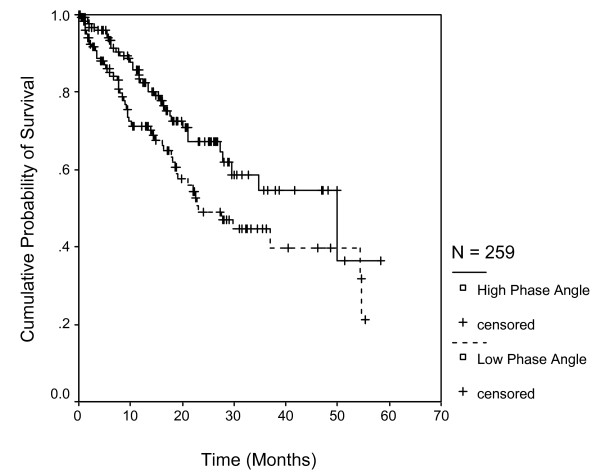
**Survival stratified by phase angle categories with cutoff of 5.6.** Each drop in a probability curve indicates one or more events in that group. Vertical lines indicate censored patients, i.e., those who reached the end of their follow-up without experiencing death.

Figure 1 shows the survival curves for the two categories of the phase angle. Patients with phase angle <= 5.6 had a median survival of 23.1 months (95% CI: 14.2 to 31.9; n = 129), while those > 5.6 had 49.9 months (95% CI: 35.6 to 77.8; n = 130); the difference being statistically significant (p = 0.031).

Table 3 summarizes the results of multivariate Cox regression analyses. Multivariate Cox modeling, after adjusting for stage at diagnosis and prior treatment history found that every one unit increase in phase angle score was associated with a relative risk of 0.82 (95% CI: 0.68 to 0.99, P = 0.041). Stage at diagnosis (p = 0.006) and prior treatment history (p = 0.001) were also predictive of survival independent of each other and phase angle. Phase angle was also used as a squared term in Cox regression due to its non-linear association with mortality (Table 4).

**Table 3:** Multivariate Cox proportional hazard model (phase angle as a continuous variable)

**Independent Variable**	**Unit of increase**	**RR^1^**	**95% CI**	**P-value**
Phase angle	1 degree	0.82	0.68, 0.99	0.041
Stage at Diagnosis	Stage I and II as referent	1.9	1.2, 2.9	0.006
Treatment History	Newly Diagnosed as referent	7.9	4.1, 15.5	0.001

**Table 4:** Multivariate Cox proportional hazard model (phase angle as a squared term)

**Independent Variable**	**Unit of increase**	**RR^1^**	**95% CI**	**P-value**
Squared Phase angle	1 degree square	0.98	0.96, 1.001	0.06
Stage at Diagnosis	Stage I and II as referent	1.9	1.2, 2.9	0.007
Treatment History	Newly Diagnosed as referent	7.9	4.1, 15.2	0.001

^1 ^Relative risk (Cox proportional hazard)

N = 259

## Discussion

The current study was undertaken to investigate if BIA-derived phase angle could predict survival in breast cancer.

This study demonstrated that phase angle is a strong predictor of survival in breast cancer after controlling for the effects of stage at diagnosis and prior treatment history. A similar study conducted in patients with advanced lung cancer stratified the patient cohort by the mean phase angle score of 4.5. Interestingly, patients with phase angle scores less than or equal to 4.5 had a significantly shorter survival than those with phase angle scores greater than 4.5 [38]. In our previous study in stage IV colorectal cancer patients, we found that phase angle above the median cut-off of 5.6 was associated with better survival [37]. Similarly, in stage IV pancreatic cancer, phase angle above the median cut-off of 5 was associated with improved survival [36].

This study adds to the growing body of evidence regarding the clinical applications of BIA derived phase angle beyond its use in body composition equations. Although the biological meaning of phase angle is not well understood, it reflects not only body cell mass, but is also one of the best indicators of cell membrane function, related to the ratio between extracellular water and intracellular water [28]. Schwenk et al. has hypothesized that phase angle could possibly be interpreted as a global marker of malnutrition in HIV infected patients [35]. In another study conducted on HIV-infected patients, it was argued that phase angle reflects the integrity of vital cell membranes [33]. In patients with liver cirrhosis, phase angle was speculated to be a marker of clinically relevant malnutrition characterized by both increased extracellular mass and decreased body cellular mass [30]. In advanced lung cancer, phase angle was speculated to be an indicator of altered tissue electrical properties [38]. In spite of lack of standardized cut-off values, phase angle seems to play an important role as a marker of morbidity and mortality in a wide range of disease conditions, with higher phase angle reflecting a general indicator of wellness [28].

Limitations of this study relate to the BIA technique and retrospective study design. This study, because of its retrospective nature, relies on data not primarily meant for research. One potential limitation of the BIA approach for estimating body composition is the reliance on regression models, derived in restricted samples of human subjects, which limits the usefulness of the derived model in other patients who differ from the original sample in which the model was developed [39,40]. However, in our study, we looked at phase angle which does not depend on regression equations to be calculated, thereby eliminating a large source of random error [3]. It has also been suggested that the variability of direct bioimpedance measures (resistance, reactance, and phase angle) depends on age, gender, and body mass characteristics of the study population which could possibly limit the extrapolation of the model [28,39,41]. A review article by Foster et al. argued that although the correlation between whole-body impedance measurements and body composition is experimentally well established, the reason for the success of the impedance technique is much less clear [42].

Some other reported limitations of using BIA for assessment of body composition are hydration status and/or major disturbances of water distribution, body position during procedure, ambient air and skin temperatures, recent physical activity, conductance of the examining table, and food consumption [43]. Since the original intent of the BIA in this study was to gather estimates of body composition as part of a baseline nutritional assessment in a clinical setting, not all of these factors could realistically be controlled. Patients were free of visible edema or ascites so there was control for obvious overhydration. Body position was controlled for because all patients were in the supine position in a bed or on an exam table. Air temperature was within a controlled range in our hospital setting. Physical activity was limited in these patients due to the advanced nature of their disease. Finally, food intake was not controlled for in this clinical setting, which may have contributed to a small amount of variability.

The cut-off point for phase angle in the present study was generated so as to divide the patient population into 2 equal and mutually exclusive groups. The cut-off value of phase angle in out study might differ from those in other patient populations. Although our cut-off point was in agreement with those reported by other studies [30,35,38], there is a clear need to define threshold values for phase angle as a nutritional assessment tool using Receiver Operating Characteristic analysis based on large prospective studies in advanced cancer. We also think that restricting the analysis to newly diagnosed patients (patients with no prior treatment history) would have been more accurate, since it would have allowed for evaluation of true overall survival time, i.e. time from the date of diagnosis to the date of death. However, doing so would have caused a significant reduction in the sample size. In our study, the survival time was calculated from the day of first visit at our hospital because the BIA measurements were not available at the time of diagnosis for previously treated patients. This limitation emphasizes the need for conducting prospective studies, which have nutritional information available since the date of diagnosis. No assessment of inter-rater reliability of the users of BIA was made in this study. This bias, however, was minimized by restricting the use of BIA to well-trained dietitians with an expertise in the use of this clinical technique.

## Conclusion

In summary, our study has demonstrated the prognostic significance of phase angle in breast cancer after controlling for the effects of stage at diagnosis and prior treatment history.

## Competing interests

The authors declare that they have no competing interests.

## Authors' contributions

DG was the main author of the manuscript, participated in concept, design, data collection, data analysis and data interpretation. CAL, JK, and SLD participated in concept, design, data collection and writing. PGV participated in concept, design and data interpretation. JFG assisted with the statistical analysis and data interpretation. CGL participated in concept, design, writing and data interpretation. All authors read and approved the final manuscript.

## Pre-publication history

The pre-publication history for this paper can be accessed here:


